# Go to the Hospital or Stay Here?: A Randomized Clinical Trial

**DOI:** 10.1093/geroni/igaa057.2742

**Published:** 2020-12-16

**Authors:** Sarah Worch, Ruth Tappen

**Affiliations:** 1 The Ohio State University, Columbus, Ohio, United States; 2 Florida Atlantic University, Boca Raton, Florida, United States

## Abstract

With the exception of guides for making end of life choices, there are very few if any patient decision aids created for residents of long-term care facilities. Further, only half of patient decision aids produced for any purpose have actually been field tested with patients and even fewer have been evaluated by providers other than the developers of the decision aid. Development of Go to the Hospital or Stay Here? was based on expert experience combined with extensive input from over 270 long-term care residents, their families and their caregivers. The initial clinical trial of this decision aid is reported in this presentation. Increased knowledge, reduced decisional conflict, increased preference for care in the nursing home when possible and a high rating of the helpfulness of the Guide were found in those who received the Guide (n=97) compared to those who did not (n=95).

